# The Epigenetic Regulation of Agronomic Traits and Environmental Adaptability in *Brassicas*

**DOI:** 10.1111/pce.70177

**Published:** 2025-09-11

**Authors:** Daolei Zhang, Yin Lu, Wei Ma, Jianjun Zhao

**Affiliations:** ^1^ State Key Laboratory of North China Crop Improvement and Regulation, Key Laboratory of Vegetable Germplasm Innovation and Utilization of Hebei, Ministry of Education of China‐Hebei Province Joint Innovation Center for Efficient Green Vegetable Industry, International Joint R & D Center of Hebei Province in Modern Agricultural Biotechnology, College of Life Sciences, College of Horticulture Hebei Agricultural University Baoding China; ^2^ Biotechnology Research Institute Chinese Academy of Agricultural Sciences Beijing China; ^3^ School of Life Science Inner Mongolia University Hohhot China

**Keywords:** agronomical traits, Brassica crops, epibreeding, epigenetic landmarks, epigenome editing, stress responses

## Abstract

As essential sources of vegetables, oilseeds, and forage, *Brassica* crops exhibit complex epigenetic regulation mechanisms involving histone modifications, DNA modifications, RNA modifications, noncoding RNAs, and chromatin remodelling. The agronomic traits and environmental adaptability of crops are regulated by both genetic and epigenetic mechanisms, while epigenetic variation can affect plant phenotypes without changing gene sequences. Furthermore, the impact of epigenetic modifications on plant phenotype has accelerated the crop breeding process. This review highlights the epigenetic mechanisms underlying agronomic and stress‐related traits in *Brassica* crops, while systematically identifying and categorising RNA modification‐associated proteins within these species. We further propose an innovative strategy for improving *Brassica* crops yield through epigenome editing technology. Finally, we discuss the prospects and challenges for the future application of epigenetics‐mediated crop breeding (epibreeding) strategies in *Brassica* crops.

## Summary statement

This review highlights the epigenetic mechanisms underlying agronomic and stress‐related traits in *Brassicas*, identifying candidate epigenetic landmarks within these species, and offering strategies for improving *Brassica* crops yield through epibreeding.

## 1 Introduction

The *Brassica* genus encompasses six *Brassica* species, including three diploid species‐*Brassica rapa* (AA genome), *Brassica nigra* (BB genome) and *Brassica oleracea* (CC genome) and three amphidiploid species‐*Brassica juncea* (AABB genome), *Brassica carinata* (BBCC genome) and *Brassica napus* (AACC genome), which collectively form the renowned “Triangle of U” (Zhang et al. 2024). Notably, *Brassica rapa* exhibits remarkable morphological diversity across its various subspecies, encompassing heading Chinese cabbage, non‐heading pak choi, enlarged turnip tubers, and the oil seed crop yellow sarson. As one of the most widely cultivated and consumed crops in the world, *Brassica* crops hold substantial agricultural and economic significance. The global market value of *Brassica* crops is projected to exceed USD 92 billion by 2022 (Zhang et al. 2024), underscoring their economic importance. Consequently, the development of high‐yielding, superior‐quality *Brassica* varieties through advanced breeding strategies is crucial for sustaining global agricultural productivity and driving economic growth.

*Brassica* crops have genetic complexity; for example, *Brassica* crops are mostly polyploidy (*Brassica napus* is allotetraploid), and the genome is large and complex. Conventional breeding approaches, which primarily depend on natural or induced genetic variation, demonstrate limited efficiency in improving complex traits, such as balancing crop yield and stress tolerance. Therefore, these constraints underscore the necessity for expanding breeding approaches to facilitate the development of high‐yield, stress‐tolerant *Brassica* varieties. In contrast to genetic variation, epigenetic variation offers a novel regulatory mechanism for crop development that operates independently of DNA sequence alterations (Mercé et al. 2020). The regulation of complex traits in crops involves a sophisticated interplay among genetic, epigenetic, and environmental factors. The environment‐induced epigenetic variation potentially accelerates plant adaptive evolution and shorten breeding cycles (G. Peng et al. 2023). For instance, salt stress can induce epigenetic alterations in *Brassica napus*, thereby regulating its adaptive mechanisms under saline condition (Guangyuan et al. 2007). A new strategy for crop breeding, epibreeding, which regulates crop agronomic traits or environmental adaptability by influencing the level of epigenetic modification in crops.

*Brassica* crops exhibit abundant epigenetic variation, which provides the potential for optimising *Brassica* crops breeding. For instance, some epilines were generated by selecting *Brassica napu* lines have demonstrated improved energy use efficiency, enhanced drought tolerance, and optimised nitrogen utilization (Verkest et al. 2015). Furthermore, epigenetic modifications throughout microspore embryogenesis in *Brassica* spp (Ahmadi et al. 2018), confirming the practicability of epibreeding in *Brassica* crops. Recent advances in plant epigenomic sequencing technologies have progressively elucidated the epigenetic landscape of *Brassica* crops, including genome‐wide profiles of histone modifications, DNA methylation, RNA modifications, chromatin remodelling dynamics, and noncoding RNA regulation. Despite these advancements, current research remains fragmented, lacking comprehensive integration of *Brassica* epigenetic regulatory networks. Notably, the development of epigenetic editing tools in *Brassica* lags behind model species like *Arabidopsis* and rice. For example, the modification of *OsFIE1* (*Oryza sativa Fertilisation‐Independent Endosperm 1*) gene by SunTag‐dCas9‐TETcd system resulted in a series of epigenetic allelic mutants with different degrees of dwarfing in rice (S. Tang et al. 2022). Therefore, excavating beneficial epigenetic variations is a novel approach to increase the yield of *Brassica* crops.

Here, we systematically summarise the key epigenetic factors influencing yield potential in *Brassica* crops and elucidate the underlying epigenetic regulatory mechanisms governing yield‐related traits and environmental adaptability. By systematically characterising RNA modification‐associated protein families in *Brassica* crops, we propose a framework to guide epibreeding‐driven enhancement of yield‐related traits and stress resilience in these species. Finally, combined with the application of epigenome editing in plants, we proposed an innovative strategy to improve the yield and environmental adaptability of *Brassica* by using epigenome editing, which opened up a novel approach for cultivating high‐yield and stresses tolerance *Brassica* crop varieties.

## 2 Histone Code Reprogramming: Enhancing Agronomic Traits and Stress Tolerance in *Brassicas*

There are many posttranslational modifications (PTMs) on histones, which can regulate chromatin status and gene expression. In plants, histone modifications are integral to numerous developmental processes, including root development, fruit ripening, embryogenesis, photosynthesis, and stress response mechanisms (F. Xu et al. 2024). These modifications are dynamically regulated by specialised protein complexes: histone methyltransferases and demethylases maintain methylation homoeostasis, while histone acetyltransferases (HATs) and deacetylase modulate acetylation levels. These regulatory proteins precisely control chromatin architecture and transcriptional activity by orchestrating the spatial distribution and quantitative levels of histone modifications across the genome. Histone methylation markers can be functionally categorised into repressive and activating marks based on their transcriptional regulatory roles. Repressive marks, including H3K9me2, H3K9me3, and H3K27me3, are predominantly localised in the region of transcriptional inactivation (D. X. Zhou 2009). Conversely, activating marks such as H3K4me3 and H3K36me3 are enriched in the region of transcriptional activation (Zhao and Zhou 2012). Different histone modifications have obvious crosstalk, enabling precise regulation of downstream target gene expression. For instance, Polycomb group (PcG) complexes mediate transcriptional repression of flower‐related genes through H3K27me3 deposition, while Trithorax group (TrxG) proteins activate the same gene set via H3K4me3 modification. Additionally, they can synergistically inhibit the expression of seed development‐related genes, demonstrating the complexity of histone modification‐mediated gene regulation (F. Xu et al. 2024).

**FIGURE 1:**
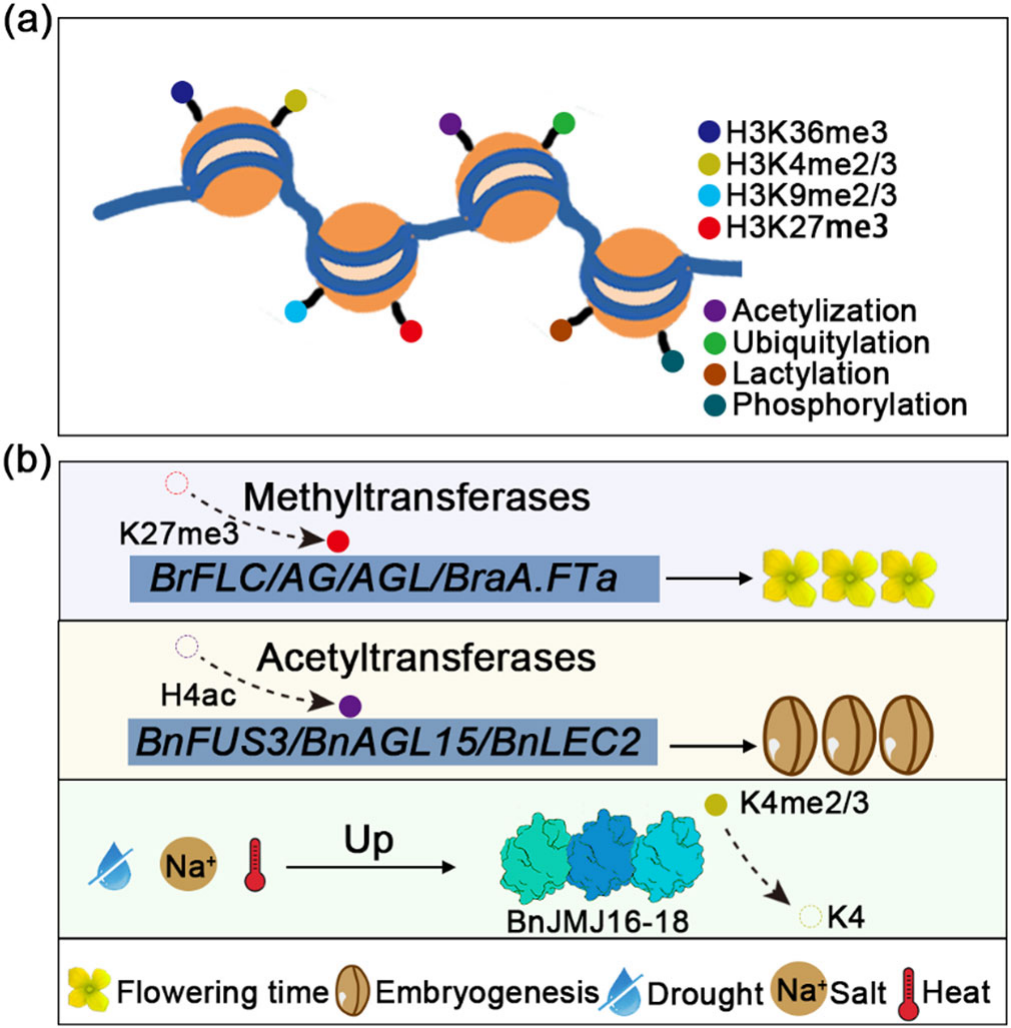
Histone modification types and functions in *Brassica* crops. (a) Currently identified histone modification types include methylation, acetylation, ubiquitylation, lactylation, and phosphorylation. (b) Histone methyltransferases mediate H3K27me3 to regulate the expression of flowering‐related genes *BrFLC*, *AG*, *AGL*, and *BraA.FTa*, thereby affecting flowering time in *Brassica* crops (Akter et al. 2019; Poza‐Viejo et al. 2024; C. Tan et al. 2021); Histone acetyltransferases mediate H4ac to regulate the expression of seed‐related genes *BnFUS3*, *BnAGL15*, and *BnLEC2*, thereby affecting embryogenesis in *Brassica* crops (Pérez‐Pérez et al. 2025); Drought, salt, and heat stress up‐regulate the expression of H3K4 demethylases BnJMJ16‐18 (BnJMJ16;a, BnJMJ17;b/c, and BnJMJ18;a), thereby affecting H3K4me2/3 levels (He et al. 2021). [Color figure can be viewed at wileyonlinelibrary.com]

*Brassica* crop is also rich in different histone modifications (Figure 1a), and related enzymes impact different developmental processes by regulating the histone modification levels of target genes (Table 1). In *Brassica napus*, *BnaSDG8.A* and *BnaSDG8.C* are homologues of the *Arabidopsis* Histone 3 lysine 36 (H3K36) methyltransferase SDG8. *BnaSDG8.A/C* can mediate H3K36me2/3 to regulate the expression of *BnaFLC* (*FLOWERING LOCUS C*) and floral transition in *Brassica napus*, Knockout of *BnaSDG8.A/C* gene by RNAi and CRISPR/Cas9 can lead to early flowering phenotype as compared with the control (Jiang et al. 2018). The SET (Su (var)3‐9, Enhancer‐of‐zeste and Trithorax) domain is a conserved motif composed of 130 amino acids, which is present in chromosomal proteins and can regulate gene expression in eukaryotes by catalysing histone methylation (Shen et al. 2025). In contrast, the JmjC (Jumonji C) regulates gene expression by histone demethylation. Together, SET and JmjC regulate various biological processes through methylation and demethylation, which are essential for maintaining proper chromatin states and gene transcription (Liu et al. 2010). In *Arabidopsis thaliana*, SET domain‐containing proteins exhibit H3K9 methyltransferase activity and catalyse H3K27 trimethylation (Hennig and Derkacheva 2009; Liu et al. 2019). Sixty genes with SET domain were identified in *B. rapa*, these genes showed random involvement in a particular organ, such as *BraSET17*, *BraSET52‐55*, and *BraSET59*. For JmiC, 59 genes were identified in *B. rapa*, these genes are also widely distributed in various tissues, and their expression also has a strong correlation, such as *BraJmjC5‐BraJmjC6*, *BraJmjC9‐BraJmjC10*, *BraJmjC34‐BraJmjC35*, and *BraJmjC40‐BraJmjC41*(Liu et al. 2019). The level of H3K27me3 varies across different developmental stages of *Brassica rapa*, exhibiting a significant negative correlation with gene expression. For instance, 2‐day‐old cotyledons display higher transcriptional activity compared to 14‐day‐old cotyledons, accompanied by lower H3K27me3 levels in gene body regions (Akter et al. 2019). Notably, *B. rapa* and *Arabidopsis thaliana* show conservation in histone markers for specific genes. For example, in all four *BrFLC* paralogs (*BrFLC1*, *BrFLC2*, *BrFLC3*, *BrFLC5*), low temperature treatment can increase H3K27me3 at the proximal nucleation site, leading to reduced *BrFLC* expression (Figure 1b) (Akter et al. 2019). Furthermore, the deletion of *BrEBM3* (*early‐bolting mutant*), which encodes the histone methyltransferase CURLY LEAF (CLF), results in an early‐bolting phenotype in Chinese cabbage. Further investigations revealed that *BrEBM3* regulates flowering time by modulating H3K27me3 deposition in *AG* (*AGAMOUS*) and *AGL* (*AGAMOUS‐like*) genes (Figure 1b) (C. Tan et al. 2021). Additionally, *BraA.REF6* (*Relative of ELF6*) and *BraA.ELF6* (*Early Flowering 6*) function as H3K27me3 demethylases in *Brassica rapa*. Intriguingly, mutations in *BraA.ELF6* lead to increased H3K27me3 levels at floral repressor *FLC* homologues, causing early flowering. Conversely, *braA.ref6* mutants exhibit delayed flowering. as a major H3K27me3 demethylase, BraA.REF6 does not regulate *B. rapa FLC* gene expression but modulates gibberellic acid (GA) biosynthetic genes via H3K27me3 (Poza‐Viejo et al. 2022). Recent studies have further identified *BraA.CLF* as a homologue of *CLF* in *Brassica rapa*, which regulates flowering time by regulating the H3K27me3 levels at flowering‐related genes, including *BraA.FTa* (*FLOWERING LOCUS T)* locus and *BraA.SOC1 (SUPPRESSOR OF OVEREXPRESSION OF CO 1)* (Poza‐Viejo et al. 2024).

**TABLE 1:** Histone‐modifying enzymes in *Brassica* crops.

Species	Enzymes	Modifiers	Functions	References
*Brassica napus*	BnaSDG8.A/C	H3K36 methyltransferases	Floral transition	Jiang et al. (2018)
	BnJMJ16‐18	H3K4 demethylase	Salt and heat stress	He et al. (2021)
	BnHAC5‐like	H4 acetyltransferase	Embryogenesis	Pérez‐Pérez et al. (2025)
*Brassica rapa*	BrEBM3	H3K27 methyltransferase	Early‐bolting	C. Tan et al. (2021)
	BraA.CLF	H3K27 methyltransferase	Flowering time	Poza‐Viejo et al. (2024)
	BraA.REF6/ELF6	H3K27 demethylases	Early flowering	Poza‐Viejo et al. (2022)
	BrHAT	H3K56 acetyltransferases	Low temperature stress	Bian et al. (2024)

Histone modifications may also play a critical role in regulating the environmental adaptability of *Brassica* crops (Figure 1b). In *Brassica napus*, for instance, several members of the *BnKDM5* (*histone lysine demethylases 5*) subfamily exhibit upregulated expression under drought, NaCl, or high temperature stress. Specifically, *BnJMJ16;a, BnJMJ17; b/c* and *BnJMJ18;a* demonstrate significantly elevated expression levels under these stress conditions (Figure 1b) (He et al. 2021). Hybrids have better phenotypes than parents under stress conditions, polyploidization of *Brassica* crops is also a model organism for studying heterosis. In *B.napus*, the variation in H3K4me3 levels is significantly positively correlated with gene expression in both parental lines and hybrids. Notably, H3K4me3 and H3K27me3 modifications exhibit greater stability during the development of *B. napus* hybrids compared to their parents. *Brassica napus* is a potentially fertile species, and histone modifications vary across different genomes. H3K4me3 and H3K9ac in the Cn subgenome show higher diurnal oscillation activity than in the An subgenome (Z. Xue et al. 2023). Furthermore, temperature fluctuations can induce genome‐wide changes in histone modification levels, leading to altered transcriptional activity of stress‐responsive genes (Hou et al. 2019). Low temperature is a pivotal factor influencing the yield of *Brassica rapa*. Histone acetylation plays a crucial role in the plant's response to low‐temperature stress, such as *BrHAT* (Histone acetyltransferases)‐related genes have changed significantly under low temperature stress (Bian et al. 2024). Moreover, stress‐induced microspore reprogramming can up‐regulate *BnHAC5‐like* and increase histone acetylation levels; suberoylanilide hydroxamic acid (SAHA), a histone deacetylase inhibitor, SAHA‐treated prombryos showed higher expression of embryogenesis transcription factors *BnFUS3* (*FUSCA3*), *BnAGL15* (*AGAMOUS LIKE15*) and *BnLEC2* (*LEAFY COTYLEDON2*) (Pérez‐Pérez et al. 2025). Additionally, Protein ubiquitination is also an important posttranslational regulatory mechanism. Plants utilise the ubiquitination pathway to adapt to changing environmental conditions. BnaJUL1 (JAV1‐associated ubiquitin ligase 1) is a RING‐type E3 ubiquitin ligase that ubiquitinates and degrades BnaTBCC1 (a tubulin‐binding cofactor C domain‐containing protein), thereby regulating drought resistance in *Brassica napus* (J. Hu et al. 2024). In short, these PTMs are essential for regulating both development and environmental adaptability in *Brassica* crops. Identifying novel histone modification‐related enzymes or developing inhibitors targeting these modifications offers promising avenues for enhancing crop resilience and productivity in the future (Table 1).

## 3 *Brassica* Traits Regulation: How DNA Methylation Shapes Agronomic Performance

Currently, over 17 distinct DNA modifications have been identified, including 5‐methylcytosine (5mC), N6‐methyladenine (6 mA), N7‐methylguanine (7mG), 5‐carboxycytosine (5cC), 5‐formylcytosine (5fC), 5‐hydroxymethylcytosine (5hmC), N4‐methylcytosine (4mC), 5‐formyluracil, 2‐aminoadenine, and N6‐carbamoyladenine (Raiber et al. 2017). These modifications are intricately regulated by a network of proteins, including methyltransferases (writers), demethyltransferases (erasers), and recognition proteins (readers), which collectively maintain precise control over DNA modification level. Among these modifications, DNA 5mC is the most prevalent and well‐studied in eukaryotes. Writers of this modification add a methyl group (CH3) to cytosine residues, altering their structure and influencing plant development and environmental adaptability. The main sequence contexts of DNA 5mC distribution are CG, CHG and CHH (H represents C, A, or T), which are mainly distributed in plant transposable elements (TEs) and gene regions, and have a significant negative correlation with gene expression. DNA 5mC methyltransferase can be divided into responsible for maintaining 5mC and *de novo* 5mC, METHYLTRANSFERASE (MET) and CHROMOMETHYLASE (CMT) are mainly responsible for maintaining 5mC levels by catalysing CG and CHG sites, while DOMAINS REARRANGED METHYLASEs (DRMs) and DRM‐like (DRML) proteins are mainly responsible for *de novo* 5mC by catalysing CHH sites (Du et al. 2015). Furthermore, DNA 5mC is closely intertwined with histone modifications. For instance, SAWADEE HOMEODOMAIN HOMOLOGUE 2 (SHH2), a recognition protein of H3K9me1, also plays a role in RNA‐directed DNA methylation pathways, contributing to the establishment of *de novo* 5mC marks (Wang et al. 2021).

*Brassica* crops exhibit a complex polyploid genome evolution. In early studies, a modified reduced representation bisulfite sequencing method was employed to generate a genome‐wide DNA methylation distribution map in *B.rapa*, revealing that 52.4% of CG sites were methylated (5mC) (Chen et al. 2015). Single‐copy genes in *Brassica rapa* display significantly higher hypermethylation levels compared to multi‐copy genes, with evolutionary differences in DNA methylation primarily driven by single‐copy genes. Furthermore, 5mC levels show a significant negative correlation with gene expression in *B.rapa* (Chen et al. 2015). Methyl‐CpG‐Binding Domain (MBD) proteins play a crucial role in plant development. *B.napus* has 22 MBD genes, its diploid ancestors, *B.rapa* and *B.oleracea* have 10 and 11 MBD genes, respectively; these MBD genes are widely expressed in flower, leaf, silique, and stem tissues (Xiao et al. 2022). The DNA methylation levels of a normal and a highly inbred degenerated variety of the Chinese cabbage (*Brassica rapa* L.ssp. *pekinensis*) were analysed. The results showed that the DNA 5mC and endogenous IAA of inbred plants were decreased, indicating that DNA 5mC may regulate the auxin pathway and lead to inbred depression phenotypes in Chinese cabbage (Liu et al. 2018). Phenotypic diversity in organisms is mostly driven by positive selection. Therefore, the analysis of positively selected genes (PSGs) provides valuable insights into the evolutionary processes of polyploids. In PSGs, methylation of CG and CHG sites predominantly occurs in introns and untranslated regions (UTRs), with methylation levels proportional to the percentage of transposons. Additionally, DNA 5mC is associated with reduced expression of PSGs (Guo et al. 2021). Analysis of DNA 5mC on homologous genes of natural allopolyploid *Brassica napus* and an in silico “hybrid” revealed that some homologous genes have asymmetric epigenetic modification and generational transmission characteristics. Additionally, the analysis demonstrated that the number of gene pairs exhibiting biased methylation levels in the C subgenome was significantly higher than in the A subgenome (M. Li et al. 2021). Polyploidization plays a pivotal role in plant evolution, and subgenome dominance is a distinct phenomenon associated with allopolyploids. This phenomenon reflects gene fractionation and expression biases across subgenomes, with genes in the dominant subgenome showing higher expression levels compared to their syntenic paralogs (homoeologs) in *Brassica* crops. While earlier research indicated that subgenome dominance correlates positively with subgenomes characterised by lower transposon densities and reduced methylation levels near genes, recent work by Zhang et al. challenges this notion. They propose a “nuclear chimera” model, suggesting that differences in transposon methylation near genes are insufficient to drive biased gene expression (Zhang et al. 2023). The SMI (SCR Methylation Inducer 1 and 2) loci, which are widely distributed in cruciferous plants, play a significant role in the transition between self‐incompatibility and self‐compatibility. Overexpression of *BnSMI‐1* in self‐incompatible *Brassica napus* (S‐70^S1300S6^) led to a notable increase in DNA 5mC levels in the promoter regions of *BnSCR‐6* and *BnSCR‐1300*, resulting in self‐incompatibility in *Brassica napus*. Furthermore, epigenome rearrangement occurred in synthetic tetraploids derived from Chinese cabbage (*Brassica rapa* L.ssp. *pekinensis*) and cabbage (*Brassica oleracea* L.var. *capitata*), and the activation of LTR/Gypsy elements in synthetic tetraploids may lead to the reduction of methylated regions (Cai et al. 2024).

DNA methylation is highly responsive to various stresses. For example, in *Brassica napus*, Huyou 2 (a heat‐tolerant genotype) and Fengyou 1 (a heat‐sensitive genotype) exhibit distinct methylation patterns under heat stress. Heat treatment significantly increases DNA methylation in Fengyou 1, while Huyou 2 undergoes more DNA demethylation events. These findings suggest that DNA 5mC plays a critical role in the thermal adaptability of *B. napus* (Gao et al. 2014). Additionally, heat stress can induce cultured microspores into embryogenesis in *Brassica napus*. For instance, treating cultured microspores of *B. napus* cv. Topas at 32 ° C for 6 h induces DNA methylation in CG and CHG contexts, providing valuable insights into the role of DNA methylation in enhancing thermal responses in *Brassica napus* (J. Li et al. 2016). Higher DNA 5mC levels may inactivate transposons and regulate the drought resistance of *Brassica juncea* (Sharma et al. 2017). Photoperiod and vernalisation are two key environmental cues regulating flowering. Methylated‐DNA immunoprecipitation sequencing (MeDIP‐seq) analysis of normal and vernalized leaves in *Brassica rapa* revealed that *BrCKA2* (*the catalytic α‐subunit of CK2*) and *BrCKB4* (*the regulatory β‐subunit of CK2*) undergo gradual demethylation during vernalisation, accompanied by increased expression. Elevated levels of *BrCKA2* and *BrCKB4* can shorten the expression cycle of *BrCCA1* (*circadian rhythm‐related 1*), further influencing flowering regulation (Duan et al. 2017). The DNA methylation levels of CG, CHG and CHH in leaves of four‐leaf stage non‐heading Chinese cabbage were 39.3%, 15.38% and 5.24%, respectively, according to the single‐base resolution methylome map, methDEGs (Differently‐expressed genes with differentially‐methylated regions in the promoter or gene body) were different in the early and late stages of heat stress (Liu et al. 2018). DNA methylases and demethylases in *B. napus* also respond to salt and heat stresses, leading to dynamic changes in DNA methylation levels. For instance, high temperature stress and salt stress can induce the expression of DNA methyltransferase‐related genes *BnaDNMT2s*, *BnaDRMa*, and *BnaDRMg*, while *BnaCMTa*, *BnaDRMd*, *BnaDRMc*, and members of the *BnaMETs* gene family showed downregulation in response to salt stress; additionally, both stresses affect demethylase gene expression, with *BnaDML3a*, *BnaROS1a*, and *BnaROS1b* downregulated under salt stress, while *BnaROS1a* expression also reduced under high temperature stress (Fan et al. 2020). High temperature (28°C or 22°C) can induce the promoter hypermethylation of ribosome biogenesis‐related genes in *Brassica oleracea*, which leads to the suppression of the apex‐highly‐expressed distinctive genes and abnormal floral development, while 5‐azacytidine (5‐azaC, a DNA methylation inhibitor) can restore the expression of these inhibitory genes (Yao et al. 2022).

In summary, DNA modifications play a crucial role in the development and environmental adaptability of *Brassica* crops. Modulating the levels of these modifications presents a promising strategy for enhancing *Brassica* crop yields. However, the distribution and function of other DNA modifications in *Brassica* crops remain poorly understood. While DNA 4mC, 6 mA and 5hmC modification have been found in *Arabidopsis*, their specific patterns and functions in *Brassica* crops remain unexplored. Deciphering DNA modification patterns and identing related DNA modification enzymes in *Brassica* crops holds the potential to improve crop productivity.

## 4 RNA Methylome Plasticity: Regulating Environment Adaptability of *Brassicas*

**FIGURE 2:**
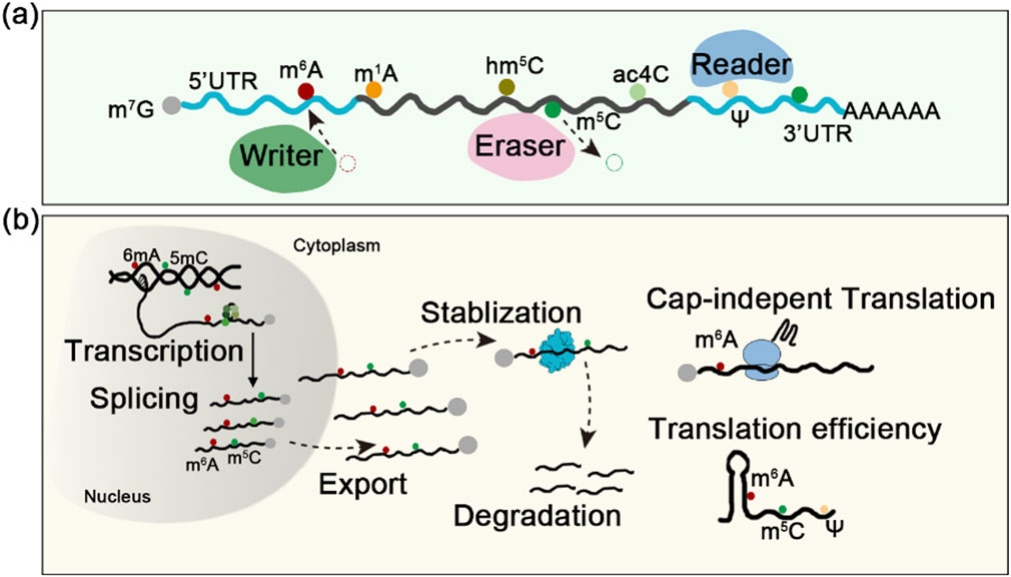
Diverse molecular functions of RNA modifications in coding RNAs. (a) Known RNA modifications in plants include transcript features such as the N7‐methylguanosine (m⁷G) cap, along with internal modifications encompassing N6‐methyladenosine (m⁶A), N1‐methyladenosine (m¹A), 5‐methylcytosine (m⁵C), 5‐hydroxymethylcytosine (hm⁵C), N4‐acetylcytidine (ac⁴C), and pseudouridine (Ψ) (D. Zhang et al. 2024; D. Zhang et al. 2022). (b) In the nucleus, DNA carries 6 mA and 5mC modifications. Post‐transcriptionally, m⁶A, m⁵C, and potentially other mRNA modifications alter pre‐mRNA splicing. Following export to the cytoplasm, a process enhanced by m⁶A and m⁵C, these mRNA modifications further modulate translation efficiency and fidelity (Gilbert et al. 2016). [Color figure can be viewed at wileyonlinelibrary.com]

Compared with DNA, RNA exhibits structural instability both in vivo and in vitro, which necessitates diverse posttranscriptional modifications to stabilise RNA metabolism in cells (Figure 2). To date, over 160 distinct RNA modifications have been identified across biological systems. Emerging evidence highlights their crucial regulatory roles in plant physiology: N6‐methyladenosine (m^6^A) modulates embryonic development, meristem differentiation, floral transition and stress response (Liang et al. 2020); N1‐methyladenosine (m^1^A) influences chlorophyll biosynthesis, while pseudouridine (Ψ) chloroplast ribosome biosynthesis and translation (Zu et al. 2023). These modifications are dynamically orchestrated by a triad of regulatory proteins: Writers (depositing modifications), Erasers (removing modifications), and Readers (recognising modification signals) (Figure 2a). Together, they form a sophisticated regulatory network that controls gene expression through multiple mechanisms, including pre‐mRNA splicing, RNA stability, subcellular trafficking, transcription elongation, and translational efficiency (Figure 2b).

The diversity of RNA modifications introduces a novel layer in regulating gene expression, giving rise to the emerging field of “epitranscriptomics.” With advancements in sequencing technologies, increasing studies are unravelling the functions of RNA modifications in crops. Among these, m^6^A is the most abundant modification in eukaryotic mRNA. MeRIP‐seq (Methylated RNA immunoprecipitation sequencing) was the first method developed to detect m^6^A sites and remains the primary detection approach. Both PA‐m^6^A‐seq (photo‐crosslinking‐assisted m^6^A sequencing) and m^6^A‐CLIP (Crosslinking immunoprecipitation‐high‐throughput‐sequencing) (Han et al. 2019) employ ultraviolet light to induce covalent cross‐linking between antibodies and RNA containing m^6^A. The m^6^A‐LAIC‐seq (m^6^A‐level and isoform‐characterisation sequencing) method identifies false positive m^6^A sites and further quantify the level of m^6^A modification in RNA (Molinie et al. 2016). Additionally, MAZTER‐seq, based on bacterial ribonuclease MazF, is an antibody‐independent method capable of quantifying m^6^A methylation at single‐nucleotide resolution (Garcia‐Campos et al. 2019). Another antibody‐independent technique, m^6^A‐REF‐seq (m^6^A‐sensitive RNA‐endoribonuclease‐facilitated sequencing), utilises the sensitivity of endonucleases to m^6^A to digest RNA fragments. DART‐seq (deamination adjacent to RNA modification targets), an antibody‐free method for global m^6^A detection, employs the fusion of cytidine deaminase APOBEC1 and the m^6^A‐binding YTH domain to induce C‐to‐U deamination at sites adjacent to m^6^A residues (Meyer 2019). The m^6^A‐label‐seq, a metabolic labelling method enabling base‐resolution identification of RNA m^6^A, locates m^6^A sites based on A‐to‐T/C/G mutation information (Shu et al. 2020). Furthermore, the FTO‐assisted chemical labelling method, termed m^6^A‐SEAL (m^6^A selective chemical labelling method), leverages the oxidation activity of FTO, a demethylase of m^6^A, for specific m^6^A detection (Wang et al. 2020). Despite extensive research into m^6^A identification techniques, several concerns persist regarding current sequencing methods. For instance, antibody‐based approaches are susceptible to nonspecific antibody binding, which can lead to inaccurate or unreliable results. Additionally, enzymatic digestion of RNA fragments introduces fixed motif effects, limiting the versatility and applicability of such methods. Furthermore, chemical labelling detection methods currently exhibit relatively low efficiency, which may hinder precise and comprehensive m^6^A profiling. These limitations underscore the need for the development of novel methodologies to advance the study of m^6^A in eukaryotes.

Advancements in sequencing technologies have significantly facilitated m^6^A research in plants, particularly in model organisms like *Arabidopsis* and rice (Liang et al. 2020). Notably, simultaneous deletion of m^6^A methyltransferases MTA, MTB, and FIP37 resulted in embryonic lethality in *Arabidopsis* (Zhong et al. 2008), underscoring the critical role of these enzymes in plant development. Similarly, the deletion of m^6^A demethylase ALKBH10B led to delayed flowering in *Arabidopsis* (Duan et al. 2017), highlighting the regulatory role of m^6^A in floral timing. However, progress in studying m^6^A in *Brassica* crops has been relatively slow. Recent investigation has begun to shed light on m^6^A dynamics in *Brassica rapa ssp. chinensis* (pak‐choi) under heat stress conditions. These studies revealed that m^6^A peaks were predominantly enriched in the 3' untranslated regions of *Brassica rapa* transcripts both before and after heat shock. Interestingly, these m^6^A modifications exhibited a certain correlation with gene expression levels, suggesting that m^6^A may play a regulatory role in the heat stress response of *Brassica rapa* (Liu et al. 2020). These findings highlight the potential importance of m^6^A in conferring stress resilience to *Brassica* crops, warranting further exploration into its mechanisms and applications.

The core components responsible for m^6^A catalytic activity are MTA and MTB, which belong to the MT‐A70 family. In cruciferous plants, MT‐A70 family proteins can be categorized into three subgroups: MTA, MTB, and MTC. Notably, MTA and MTB are ubiquitously present across all examined cruciferous plant species, with the exception that MTC is absent in *Brassica rapa* and *Raphanus sativus*, including Chinese cabbage, pak‐choi, and yellow sarson (Supporting Information Table 1). Additionally, *Brassica napus* contains a higher number of MTC homologous proteins. ALKBH encodes an RNA m^6^A demethylase that plays a crucial role in regulating fruit ripening and environmental adaptability (Miao et al. 2020; L. Zhou et al. 2019). In *Arabidopsis thaliana*, the m^6^A erasers ALKBH9B and ALKBH10B are members of the ALKBH family, characterised by the presence of a 2‐OG‐FeII domain. In *Brassica* crops, ALKBH9A/B/C exhibit a higher number of homologous proteins, whereas ALKBH10A/B/C are relatively fewer. This suggests that ALKBH9 may play a more critical functional role in *Brassica* species (Supporting Information Table 1). RNA m^6^A readers are capable of recognising m^6^A modifications and mediating m^6^A to exert biological functions. In plants, several classes of m^6^A readers have been identified, including ECT (EVOLUTIONARILY CONSERVED C‐TERMINAL REGION) proteins, also known as YT521‐B homology‐domain proteins, which belong to the YTH family. We conducted a systematic analysis of the evolution of ECT family in cruciferous plants, including *Brassica* crops, and discovered that ECT2 is universally present across all examined cruciferous plant species (Supporting Information Table 1). As the primary m^6^A reader in plants, ECT2 plays a pivotal role in controlling developmental timing and morphogenesis in *Arabidopsis*. The widespread distribution of ECT2 homologous proteins in *Brassica napus* suggests a potential role for m^6^A in regulating the development of this crop.

M^5^C is a cytosine methylation modification commonly found in tRNA, rRNA, and mRNA. Among the methods used to detect m^5^C, m^5^C‐RIP‐seq stands out as a widely adopted approach based on m^5^C‐specific antibodies. This technique has been successfully applied in bacteria, archaea, yeast, mammals, and plants. Another powerful method for detecting m^5^C in RNA is Bisulfite‐seq. In this approach, bisulfite treatment converts unmodified cytosines (C) to uracil (U), while m^5^C remains resistant to this conversion. This allows for single‐base resolution detection of m^5^C modifications (Y. Tang et al. 2020). Aza‐IP (5‐azacytidine‐mediated RNA immunoprecipitation) represents another innovative strategy for identifying m^5^C modifications. This method exploits the covalent bond formed between RNA methyltransferases and the cytidine analog 5‐azacytidine to enrich for m^5^C‐modified RNAs (Khoddami and Cairns 2013). Additionally, m^5^C‐miCLIP (methylation‐iCLIP) has emerged as a highly specific technique for mapping m^5^C sites across the transcriptome, enabling precise identification of methylation events at the single‐base level. The release of methylated RNAs depends on a conserved cysteine residue (C271) located at position 271 of the m^5^C methyltransferase NSUN2. Mutating this residue to alanine (C271A‐NSUN2) stabilises the enzyme's interaction with RNA, thereby enhancing the detection signal at m^5^C sites (Hussain et al. 2013). In plants, m^5^C modifications exhibit sensitivity to various stress responses. For instance, treatments with auxin, cytokinin, and abscisic acid (ABA) have been shown to increase m^5^C levels, while high temperatures or drought stress reduce them (X. Cui et al. 2017). Furthermore, m^5^C plays a significant role in diverse developmental processes in plants, including root development and photosynthesis (Cui et al. 2017; David et al. 2017; Zhang et al. 2022).

The tRNA‐specific methyltransferase 4B (TRM4B), a member of the RNA (C5‐cytosine) methyltransferase (RCMT) family, catalyses m^5^C in plants. In *Arabidopsis thaliana*, the RCMT family comprises TMRM4A‐4H, with TRM4A and TRM4B being ubiquitously present across all examined cruciferous plant species. Notably, the number of TRM4B homologous proteins in *Brassica* crops exceeds that of TRM4A, consistent with previous findings that TRM4B serves as a key regulatory protein for m^5^C modifications (Supporting Information Table 2). YBX1, a human m^5^C recognition protein (Wang et al. 2023) belonging to the YBX1 family, contains a Cold Shock Domain (CSD). While m^5^C recognition proteins have yet to be identified in plants, the CSD functional domain is widely present in *Brassica* crops (Supporting Information Table 2). Interestingly, Ten‐eleven translocation protein 1 (TET1), a mammalian m^5^C eraser (Yang et al. 2022) has no homologous gene in *Brassica* crops. This suggests that the RNA m^5^C metabolic pathway in *Brassica* crops may differ significantly from that in mammals.

In brief, RNA modifications play a critical role in plant development and stress responses. However, the distribution and functions of RNA modifications in *Brassica* crops remain largely unexplored. Identifying the associated modification writers, erasers and readers could provide valuable insights and support for the future application of epibreeding in *Brassica* crops.

## 5 Multi‐Layered Epigenetic Landscapes: Chromatin States and Noncoding RNA Networks in *Brassicas*

**FIGURE 3:**
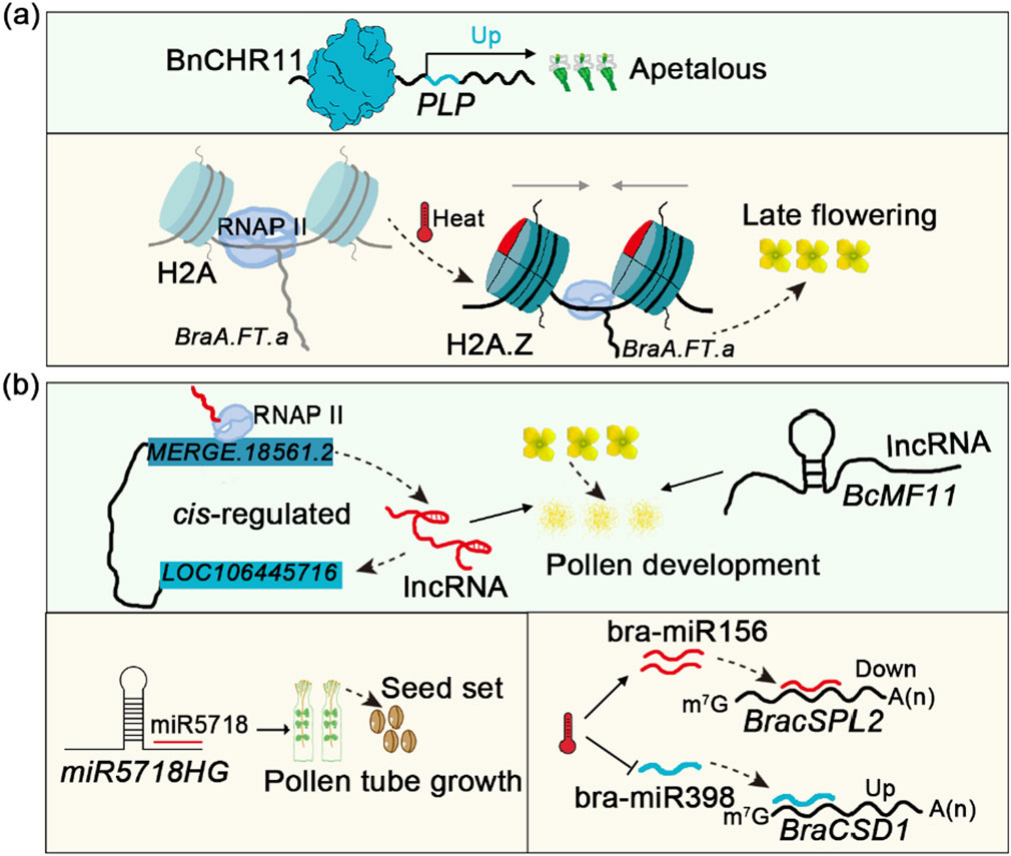
Chromatin remodelling and noncoding RNAs orchestrate diverse developmental programmes and environmental adaptability in *Brassica* crops. Specifically, BnCHR11, a SWI/SNF family member, regulates the apetalous trait in *Brassica napus* by upregulating *PLP* gene expression (K. Yu et al. 2018). High temperatures promote H2A.Z deposition on *BraA.FT.a*, thereby modulating flowering time in *Brassica rapa* (Del Olmo et al. 2019). (b) *LOC106445716* was cis‐regulated by lncRNA *MERGE.1856.1.2*, thereby affecting pollen development in *B. napus* (Xing et al. 2023); *BcMF11* is a lncRNA associated with pollen development in *Brassica campestris* (Song et al. 2013). miR5718 regulates pollen tube growth and seed set in *Brassica campestris* by targeting the *miR5718HG* gene (D. Zhou et al. 2022). Heat stress induced bra‐miR156h/g, downregulating its target gene *BracSPL2*; concurrently, it inhibited bra‐miR398a/b, upregulating the target gene *BraCSD1* (X. Yu et al. 2012). [Color figure can be viewed at wileyonlinelibrary.com]

Dynamic alterations in chromatin structure play a critical role in regulating gene transcription. ATP‐dependent chromatin remodelling factors facilitate nucleosome repositioning and histone covalent modification during gene replication and chromatin rearrangement, thereby enabling chromatin remodelling in eukaryotes (Centore et al. 2020). The SWI/SNF complex is a key chromatin remodelling factor that is highly conserved across eukaryotes, which is essential for regulating plant stress responses (Buszewicz et al. 2016; Plskova et al. 2024). Sucrose nonfermenting 2 (SNF2), a core component of the SWI/SNF complex, interacts with actin‐related proteins, SANT, and SWI proteins to form a functional complex. In plants, the SWI/SNF complex can associate with the transcriptional coactivator ANGUSTIFOLIA3/GRF‐INTERACTINGFACTOR1 (AN3/GIF1), which binds to DNA to regulate gene expression (Nelissen et al. 2015). Qian et al. identified a total of 405 putative *Snf2* genes in *Brassica* crops, including 5350 and 46 in *Brassica rapa*, *Brassica nigra*, and *Brassica oleracea*, respectively, and 9391 and 72 in *Brassica juncea*, *Brassica napus*, and *Brassica carinata*, respectively. These genes exhibited highly conserved chromosome distribution and gene structure across *Brassica* crops, with no significant dynamic changes observed during polyploidization (Qian et al. 2024). Deletion of *Arabidopsis thaliana IMITATION SWITCH* (*AtISWI*) genes, such as *CHROMATIN REMODELING11* (*CHR11*) and *CHR17*, results in a dramatic acceleration of the vegetative‐to‐reproductive transition in plants (Qian et al. 2024). In *Brassica napus*, *CHR11* has been shown to activate the expression of the *PLURIPETALA* (*PLP*) gene, which controls apetalous trait (Figure 3a) (K. Yu et al. 2018). Plant homeodomain (PHD) finger proteins also play a critical role in chromatin remodelling. These proteins regulate the expression of target genes by binding to histone modifications, either activating or repressing their transcription. Alam et al. identified 145 putative PHD finger proteins in *Brassica rapa*, with the majority predicted to be involved in protein binding activity (91.3%). Additionally, nine PHD finger genes were found to respond to drought and salt stress, including *Bra001393*, *Bra016698*, *Bra017415, Bra026210, Bra026825, Bra034169, Bra034860, Bra034950* and *Bra036568*. Chromatin remodelling also influences cuticular lipid deposition. For example, the potential chromatin remodelling factor Bol026949 is thought to regulate the Glossy Green Trait in *Brassica oleracea* (Wang et al. 2023). Furthermore, chromatin remodelling contributes to the environmental adaptability of *Brassica* crops. For instance, chromatin remodelling complex can promote thermomorphogenesis by facilitating H2A.Z eviction in *Arabidopsis* (Xue et al. 2021). In *Brassica rapa*, high temperatures increase H2A.Z levels, and elevated H2A.Z deposition on *BraA.FT.a* (*BraA02g016700.3C*) correlates with temperature‐responsive variations in flowering time (Figure 3a) (Del Olmo et al. 2019). Remarkably, while the relationship between high temperature‐regulated H2A.Z presence and *BnaFTA2* repression remains unclear across different *B. napus* accessions, H3K4me3 is an active mark closely coupled with *BnaFTA2* expression in high‐temperature flowering in late accessions (Abelenda et al. 2023). In conclusion, chromatin remodelling complexes are integral to the growth, development, and stress responses of *Brassica* crops. Their roles in regulating gene expression and environmental adaptability underscore their importance in agricultural applications.

**FIGURE 4:**
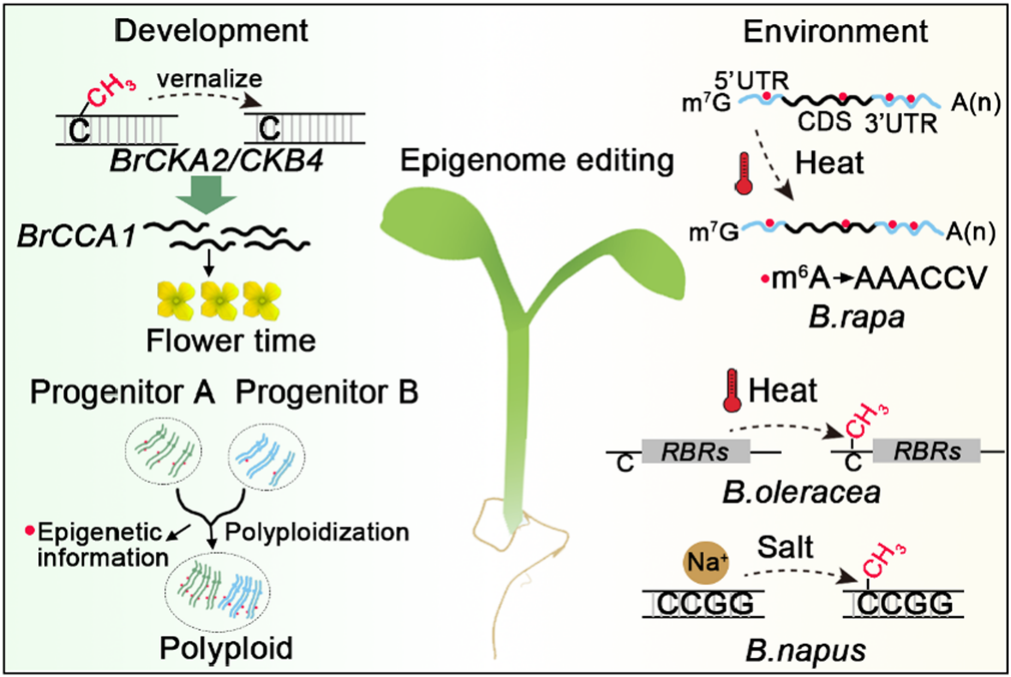
Schematic diagrams showing the ways for improving *Brassica* crops yields through epigenetic regulations. Vernalisation induces demethylation of *BrCKA2* and *BrCKB4*, thereby regulating the expression cycle of *BrCCA* to control flowering time in *Brassica rapa* (W. Duan et al. 2017). Epigenetic modifications play a key role in polyploid genome evolution. In *B. napus*, hybrids exhibit more stable levels of H3K4me3 and H3K27me3 than their parents (Z. Xue et al. 2023). Allopolyploids harbour unique epigenetic profiles, and during polyploidization, single‐copy genes in *Brassica rapa* display significantly higher hypermethylation levels compared to multi‐copy genes (Chen et al. 2015). In *Brassica rapa*, m⁶A is predominantly enriched in the 3'UTR of mRNAs both before and after heat shock, and correlates with gene expression; its recognition motif is primarily AAACCV (V: U/A/G) (G. Liu et al. 2020). Heat stress induces promoter hypermethylation of *ribosome biogenesis‐related genes* (*RBRs*) in *Brassica oleracea*, thereby impairing developmental processes (Yao et al. 2022). Additionally, salt treatment triggers methylation at CCGG sites in *Brassica napus* (Guangyuan et al. 2007), highlighting the potential of epigenetic editing for tailoring stress resilience and agronomic traits in *Brassicas*. These examples collectively underscore the regulatory versatility of epigenetic modifications in fine‐tuning gene expression networks to optimise *Brassica* growth and stress adaptation; future efforts could leverage epigenome editing to engineer these epigenetic variations, enabling balanced developmental and environmental adaptation in *Brassica* crops. [Color figure can be viewed at wileyonlinelibrary.com]

Alongside mRNA, a substantial amount of noncoding RNAs (ncRNAs) are also enriched in eukaryotes and play crucial regulatory roles in gene expression networks (Figure 4) (Guo et al. 2023). In *Brassica* crops, ncRNAs have been implicated in the regulation of key developmental traits, with numerous tissue‐specific ncRNAs being identified. Through small RNA deep sequencing analysis, Kim et al. characterised the miRNA profiles across various tissues (seedlings, roots, peduncles, leaves, and flowers) of *B. rapa*, identifying 216 novel and 196 conserved miRNAs that collectively target approximately 20% of the coding sequence regions (Kim et al. 2012). *BcMF11 (Brassica campestris Male Fertility 11)*, a noncoding RNA gene essential for pollen development in *Brassica campestris*. Antisense‐mediated suppression of *BcMF11* significantly reduces pollen germination efficiency and delays pollen tube elongation in Chinese cabbage (Figure 3b) (Song et al. 2013). Similarly, overexpression of bra‐miR5718HG impairs pollen tube growth and ultimately affects seed set in *B. campestris* (Figure 3b) (D. Zhou et al. 2022). The cytoplasmic male sterility (CMS) system represents a pivotal breeding strategy in *Brassica* crops. Comparative analysis of two CMS materials in *Brassica* napus (Nsa CMS 1258A and Pol CMS P5A) revealed 227 and 116 long noncoding RNAs (lncRNAs), respectively. Notably, *LOC106445716*, encoding β‐d‐glucopyranosyl abscisate β‐glucosidase, was cis‐regulated by lncRNA *MERGE.1856.1.2*, thereby affecting pollen development in *B. napus* (Figure 3b) (Xing et al. 2023). A high oleic acid content is an important feature of rapeseed breeding. Wang et al. investigated the dynamic expression patterns of lncRNAs during seed development in high‐ and low‐oleic‐acid *B. napus* varieties. Their findings suggest that lncRNA‐mRNA regulatory modules, particularly those involving lipid metabolism genes such as *3‐ketoacyl‐CoA synthase 16* (*KCS16*) and *acyl‐CoA:diacylglycerol acyltransferase 1* (*DGAT1*), play crucial roles in seed oil composition (Wang et al. 2023).

NcRNAs also mediate stress responses in *Brassica* species. Heat stress treatment in *B. rapa* induced dynamic changes in 4594 lncRNAs, with 25 showing strong correlations with heat shock gene expression. Functional analysis revealed that LncRNA (TCONS_00048391) modulates the heat tolerance through regulation of *bra‐miR164a* expression (Wang et al. 2019). Similarly, drought‐responsive lncRNAs have been identified in *B. napus*, with drought‐tolerant genotype Q2 exhibiting 145 lncRNA‐mRNA network nodes compared to 305 in drought‐sensitive Qinyou8 Key stress‐responsive lncRNAs, including XLOC_052298, XLOC_0094954, and XLOC_012868, were found to co‐express with stress‐related genes (X. Tan et al. 2020). Furthermore, lncRNAs on chromosome BnaA03 have been implicated in clubroot resistance in *B. napus* (Summanwar et al. 2021).

MicroRNAs (miRNAs) represent another crucial class of regulatory molecules in *Brassica* stress responses. In *Arabidopsis*, miRNAs primarily mediate gene silencing during abiotic stress responses. Yu et al. demonstrated that heat stress (46°C for 1 h) in non‐heading Chinese cabbage (*B. rapa ssp. chinensis*) regulated the expression of specific miRNA family members, with heat stress inhibiting the expression of bra‐miR398a/b, which led to increased expression of the bra‐miR398 target gene *BraCSD1*, while heat stress induced the expression of bra‐miR156h/g, which resulted in downregulation of the bra‐miR156 target gene *BracSPL2*, indicating that these specific miRNAs may regulate heat response in *B. rapa* by targeting heat stress‐related genes (Figure 3b) (X. Yu et al. 2012). Cold stress responses in *B. napus* involve upregulation of conserved miRNAs (miR164d, miR167d, miR168b, miR395c/e, miR398‐5p, and miR5717) (Megha et al. 2018), while miR18851 regulates low‐temperature sensitivity through cleavage of target transcripts *Bn.TIR.A09* (*Toll/Interleukin‐1 receptor*) and *Bn.TNL.A* (*TIR‐NBS‐LRR, TIR‐nucleotide‐binding site‐leucine‐rich repeat*) (P. Xu et al. 2023). The role of ncRNAs extends to agricultural applications, as evidenced by their involvement in herbicide resistance. Tolerant genotype M342 of *B. napus* exhibits nearly double the number of miRNAs compared to sensitive genotype N131 following sulfonylurea herbicide application (309 vs. 164) (M. Hu et al. 2017). Additionally, cis‐natural antisense transcripts (cis‐NATs) and cis‐NATs‐derived small interfering RNAs (nat‐siRNAs) participate in various developmental and stress responses in *B. rapa*, with 12 cis‐NATs showing heat stress responsiveness (X. Yu et al. 2013). Branch angle is a component of plant architecture, auxin‐ and brassinosteroid (BR)‐related pathways are the main pathways regulating branch angle. In oilseed rape (*B. napus L*.), miRNAs (miR156, miR172, miR160, miR165, and miR319) potentially regulate branch architecture through modulation of ARF and IAA‐related gene family expression (Cheng et al. 2017). In *B. napus*, miR1885 can directly silence the TIR‐NBS‐LRR class of *R* gene *BraTNL1* and inhibit the expression of photosynthesis‐related gene *BraCP24* by targeting the *Trans‐Acting Silencing* (*TAS*) gene *BraTIR1*, indicating the potential of miRNAs in the application of crop yield and disease resistance balance (C. Cui et al. 2020). Emerging evidence also implicates specific siRNAs influence the response of *Brassica* crops to viral stress (Leonetti et al. 2021), Circular RNAs (circRNAs), a class of noncoding RNA molecules, also have a potential role in *Brassica napus* response to stress (Guo et al. 2024).

## 6 Epigenome Editing: Rewiring Epigenetic Modifications Through CRISPR Systems

The evolution of gene editing technologies has revolutionised crop breeding strategies, progressing from early zinc finger nucleases and transcription activator‐like effector nucleases to the current state‐of‐the‐art CRISPR‐Cas9 system. Building upon these advancements, epigenome editing has emerged as a novel strategy for crop improvement. Epigenetic modifications play crucial roles in regulating gene expression and can be stably inherited across generations, establishing epigenetic memory that maintains both genomic stability and genetic diversity (Bonasio et al. 2010).

Traditional CRISPR‐Cas9 editing achieves crop improvement through direct DNA sequence alterations, whereas epigenome editing modulates crop yield traits and environmental adaptability by reprogramming epigenetic information without altering the underlying DNA sequence. This approach provides an innovative alternative for crop breeding by rewriting the epigenetic landscape to fine‐tune gene expression levels. The stability and heritability of epigenetic modifications make this strategy particularly promising for crop improvement (Dhakate et al. 2022). The molecular basis of epigenome editing lies in the precise manipulation of epigenetic marks, primarily through the regulation of methyltransferase and demethylase activities. By engineering editing systems that fuse these enzymatic activities with targeting modules, researchers can achieve specific activation or repression of target genes. This principle has been successfully implemented in the development of CRISPRoff and CRISPRon systems, which represent a significant breakthrough in epigenetic editing technology. The CRISPRon system utilises the 5mC demethylase TET1 to remove methyl groups, thereby activating gene expression. Conversely, the CRISPRoff system employs DNA methyltransferases (DNMT3a and DNMT3L) coupled with the KRAB repressive domain to establish methyl marks, effectively silencing target genes (Nuñez et al. 2021). Remarkably, the CRISPRoff system demonstrates exceptional efficiency, achieving stable gene silencing in 80%–90% of targeted genes (Moussa et al. 2021).

Epigenome editing breeding represents a transformative approach to enhance crop yield traits and environmental adaptability through precise modulation of epigenetic modification levels and distribution patterns. A compelling example is the expression of FTO, a human RNA m^6^A demethylase, in rice and potato systems, which reduces m^6^A levels and enhances chromatin accessibility. This modification results in significant phenotypic improvements, including root system expansion, increased photosynthetic efficiency, and enhanced drought tolerance (Q. Yu et al. 2021). The development of Cas12j2‐driven 5mC epigenetic editing systems has enabled precise regulation of key agronomic genes in rice, such as the targeted downregulation of the starch synthase gene *OsGBSS1*(Liu et al. 2022). Furthermore, the fusion of dCas9 with histone acetyltransferase HAT1 has been shown to upregulate drought‐responsive element *AREB1/ABF2* expression, thereby reducing drought sensitivity in *Arabidopsis* (de Melo et al. 2020). The regulatory potential of promoter methylation in crop improvement is well‐documented. Hypermethylation events near gene promoters can significantly influence plant growth and development. For instance, *DREB1A* promoter hypermethylation modulates plant responses to cold stress (Kidokoro et al. 2020), while methylation level variations in the *ZmCCT9* (*CO‐like Carbon cycle 9*) promoter region affect maize flowering time and plant height (Y. Peng et al. 2019). These findings underscore the importance of elucidating epigenetic modification patterns at key gene loci, which not only reveal fundamental molecular mechanisms of trait formation but also provide innovative strategies for crop improvement.

Despite significant progress in other crops, the epigenetic landscape of *Brassica* species remains relatively unexplored, particularly regarding conserved RNA modifications, such as m^6^A and m^5^C (Figure 4). The application of epigenome editing technologies in *Brassica* crops holds immense potential, as evidenced by salt‐induced methylation changes (0.2%–17.6% increase at CCGG sites) in *Brassica napus* under 10–200 mmol/L NaCl treatment (Figure 4) (Guangyuan et al. 2007). Future applications of epigenome editing tools to modify these sites could revolutionise salt tolerance in *Brassica* species (Figure 4). The unique genomic architecture of *Brassica* crops, characterised by extensive polyploidy and genome duplication events, presents both challenges and opportunities for epigenome editing applications. The complex epigenetic regulatory networks in *Brassica* species offer multiple targets for precision editing, particularly in: (1) Yield Enhancement, targeting epigenetic markers associated with flowering time regulation and biomass accumulation; (2) Stress Adaptation, modifying stress‐responsive epigenetic signatures to improve drought, salinity, temperature and pathogen tolerance; (3) Quality Improvement, reprogramming epigenetic marks linked to oil composition and nutrient content. The development of *Brassica*‐specific epigenome editing tools, combined with advanced genome‐wide epigenetic profiling, will enable precise manipulation of these traits while maintaining genetic diversity. This approach offering a novel pathway for sustainable crop improvement in the face of climate change and increasing food demand.

## 7 Summary and Recommendations

*Brassica* crops are highly valued for their nutrient‐rich composition and versatile uses as oil crops, vegetables, and condiments, making them widely cultivated worldwide. Enhancing production efficiency has consistently been a primary objective in *Brassica* breeding programmes. This review focuses on the distribution of epigenetic factors and information in *Brassica* crops; these findings underscore the potential of epibreeding as a novel strategy to improve agronomic traits and environmental adaptability (Figure 4).

The practical application of epibreeding in *Brassica* crops remains challenging due to several limitations and gaps in our understanding of epigenetic mechanisms in these species. (i) The lack of comprehensive epitranscriptomics data hinders our ability to fully understand and utilise epigenetic variations for crop improvement; (ii) Epigenetic variations are highly sensitive to environmental conditions, making it difficult to consistently identify and propagate beneficial epigenetic traits; (iii) The diversity of epigenetic modifications makes it more complicated to mine epigenetic markers associated with specific traits. (iiii) Stability and heritability of epigenetic modifications, many epigenetic changes are transient and may not persist through successive plant lifecycles; (iiiii) The existing epigenome sequencing techniques are facing labour‐intensive and costly, limiting their use in breeding programmes. To overcome these challenges and fully harness the potential of epibreeding in *Brassica* crops, the following strategies could be pursued: (1) Conduct large‐scale epigenomic studies in *Brassica* crops to map profiles of epigenetic information. (2) Identifying stable epigenetic markers that are less susceptible to environmental fluctuations. This could involve screening for epigenetic modifications that remain consistent across different growing conditions. (3) Combine epigenomic data with transcriptomic, proteomic, and metabolomic datasets to gain a holistic understanding of how epigenetic variations influence plant phenotypes. This integrated approach would help pinpoint causal relationships between specific epigenetic marks and desirable traits. (4) Utilise emerging CRISPR‐based tools, such as CRISPR‐dCas9 systems, to precisely manipulate epigenetic states of different growth stages. (5) Develop cost‐effective and high‐throughput screening methods to assess epigenetic stability and trait association. While the application of epibreeding in *Brassica* crops faces significant challenges, ongoing advancements in epigenetics research and technological innovations offer promising solutions.

## Conflicts of Interest

The authors declare no conflicts of interest.

## Supporting information

Supplementary Table 1.

Supplementary Table 2.

supmat.

## Data Availability

The data that support the findings of this study are openly available in Brassicaceae Database at http://brassicadb.cn.
