# Supplementary material for: The Epigenetic Regulation of Agronomic Traits and Environmental Adaptability in Brassicas
Source: Plant Cell Environ. 2025 Sep 11;48(12):8915–27. doi: 10.1111/pce.70177 (PMC12586914; doi:10.1111/pce.70177)
Supplement: Supplementary file 3 — supmat. [file PCE-48-8915-s002.docx]

Supplementary Table 1. The distribution of RNA m6A-associated proteins in Brassica crops.

Supplementary Table 2. The distribution of RNA m5C-associated proteins in Brassica crops.
